# Surfactin: a novel *Aphis gossypii* killing surfactin produced by *Bacillus australimaris* TRM82479 of Taklamakan Desert origin

**DOI:** 10.3389/fmicb.2025.1559495

**Published:** 2025-03-12

**Authors:** Yelin Wang, Zhibin Sun, Shiyu Wang, Feng Wen, ZhanFeng Xia

**Affiliations:** ^1^Key Laboratory of Conservation and Utilization of Biological Resources in the Tarim Basin, Alar, China; ^2^College of Life Science and Technology, Tarim University, Alar, China

**Keywords:** *Bacillus*, *Aphis gossypii*, insecticidal activity, surfactin, Taklamakan Desert

## Abstract

**Introduction:**

The cotton aphid *Aphis gossypii* poses a global, serious threat to cotton yield and quality. Although chemical pesticides are effective, pollution and resistance are increasingly prominent, making development of new biopesticides a priority in the context of green agricultural development.

**Methods:**

Given that reports on the activity of surfactins against *A. gossypii* are limited, here, 107 *Bacillus* strains isolated from the extreme environment of the Chinese Taklamakan Desert were screened for insecticidal activity against *A. gossypii* using the leaf-dip method. Active strains were characterized by morphological observation, 16S rRNA gene sequencing, and phylogenetic analysis. Secondary metabolite synthesis genes were identified by whole-genome sequencing and antiSMASH analysis.

**Results:**

*B. australimaris* strain TRM82479 showed 75.00% 48-h mortality against *A. gossypii*. An antiSMASH analysis showed that this strain contains several gene clusters related to the synthesis of nonribosomal peptide (NRP) fengycin and lichenysin lipopeptide analogs. Cluster 1 has the highest similarity of 52% with the fengycin synthesis gene cluster, and Cluster 8 has the highest similarity of 92% with the lichenysin synthesis gene cluster. It is inferred that *B. australimaris* strain can produce lipopeptide analogs distinct from fengycin and lichenysin, so we isolated and identified its NRPs. The results showed that surfactin is the main insecticidal substance, with an LC_50_ of 0.857 mg/mL and an LC_95_ of 4.350 mg/mL against cotton aphids in aqueous solution. The results of the zebrafish acute toxicity experiment showed that surfactins are low-toxic to fish, indicating good biological safety.

**Discussion:**

This study not only provides new strain resources for cotton aphid control but also demonstrates the potential of surfactins as biopesticides, laying a foundation for their future agricultural application.

## 1 Introduction

Cotton (Quan and Wu, 2023; Zhang et al., 2023) is an important global cash crop in 80 countries with a total planted area of 32 million hectares and an estimated annual value of $5.7 billion, with China, India and the United States of America the largest producers, with more than half of global production (Vitale et al., 2024). Pests are a serious threat to production; according to the United Nations Food and Agriculture Organization, annual global economic losses caused by pests are estimated to be billions of dollars (Sharma et al., 2017).

There are a wide variety of cotton pests, including *Aphis gossypii*, *Tetranychus cinnabarinus*, and *Thrips tabaci.* Currently, pest control relies primarily on chemical pesticides, which achieve quick and effective control and reduce losses; for example, synthetic pyrethroids and organophosphates significantly reduce pest populations in the short term (Malinga and Laing, 2024). Although chemical pesticides remain important, their misuse has sparked widespread controversy (Aktar et al., 2009). Excessive use of insecticides can have a negative impact on beneficial insects and the environment (Machado et al., 2019). Prolonged use of synthetic pesticides may also lead to the development of resistance (Kone et al., 2018). For these reasons, biopesticides are becoming increasingly important in the context of rising demand for green agriculture (Samada and Tambunan, 2020). Their use not only significantly reduces pollution and harm to agricultural products, but also counteracts the problem of resistance to traditional chemical pesticides (Fenibo et al., 2021).

*Bacillus* produce a variety of biologically active substances with high bacteriostatic activities, antagonistic spectra, environmental friendliness, and other advantages. They provide a powerful alternative to chemical fertilizers and pesticides, as many bacteria themselves have strong resistance, high survival, and rapid reproduction; therefore, biological control is widely used (Prasad et al., 2023). *B. thuringiensis* produces δ-endotoxins, including Cry and Cyt proteins, which are toxic to a wide range of insects. Cry proteins are effective against specific insect orders, including Lepidoptera, Diptera, Hymenoptera, and Coleoptera; whereas Cyt proteins are toxic to Diptera (Rajashekhar et al., 2017). *B. sphaericus* is capable of producing a variety of lipopeptides with antimicrobial activity and has been used to control pests. For example, lipopeptide metabolites produced by *B. subtilis* have been used to control *Spodoptera littoralis*, *Drosophila melanogaster*, *Culex quinquefasciatus*, *Anopheles stephensi* and *Aedes aegypti* (Fathy et al., 2024). Bora et al. identified compounds produced by *Bacillus* species using liquid chromatography-mass spectrometry (LC–MS), including Brevianamide A, Heptadecanoic acid, Thiolutin and Versimide, which showed toxicity against *Oligonychus coffeae* (Bora et al., 2023).

As the largest desert in China, the extreme environment of the Taklamakan Desert has created unique microbial resources, with *Bacillus* spp. the dominant bacteria (Wen et al., 2024). In this study, we aimed to screen strains with efficient *A. gossypii* killing activity and to explore their insecticidal active components to provide strain resources and theoretical support for the development of insecticidal biopesticides. To this end, we screened 107 such strains from the Taklamakan Desert for *A. gossypii* killing activity. We further characterized them by multiple methods.

## 2 Materials and methods

### 2.1 Bacterial strain

All 107 strains of *Bacillus* spp. selected for this study were isolated from the Taklamakan Desert and are preserved in the Microbial Strain Resource Bank of Tarim University using the freeze-drying method at a storage temperature of 5°C.

### 2.2 Insects

*A. gossypii* was collected from the Horticultural Experiment Station of Tarim University (81°17′E, 40°32′N) during the infestation period. For their maintenance and expansion, cotton seeds were first soaked in pure water for 2–3 days until white shoots developed. Soil was prepared from ratio of nutrient soil: coconut bricks: vermiculite at a ratio of 5:3:2. Seeds were sown at 3–5 cm in this soil and kept moist. Seedlings were watered twice or thrice weekly after emergence. After 2–3 weeks, insects were placed onto the back of the leaves using a fine brush. Insects were reared at 25°C, 50.00% humidity, and 12:12-h light: dark cycles in 50 × 50 × 65 cm (L × W × H) cages.

### 2.3 Screening

For shake-flask fermentation, bacteria were thawed at 4°C, inoculated onto LB plates, and incubated at 28°C incubators for 2 days. Colonies were inoculated into liquid LB and shaken at 120 rpm at 30°C for 2–3 days.

*A. gossypii* killing was measured from these cultures using the leaf-dip method (da Silva Queiroz et al., 2020). LB without bacteria was used as a negative control and 0.1 g/mL of a 20.00% fludioxonil suspension (Jiangsu Keshen Group Co., Ltd.) was used as a chemical control. Assays were performed in triplicate. Twenty test insects were tested per replicate. Mortality and corrected mortality rates were calculated as:


$$\begin{array}{l} \mathrm{Mortality}\;\mathrm{rate}\\ =\mathrm{number of dead insects}/\mathrm{total number of test insects}\times 100\% \end{array}$$

$$\begin{array}{c} \mathrm{Corrected}\;\mathrm{mortality}\;\left(\%\right)=\mathrm{Mortality in treatment}\;\left(\%\right)\\ -\mathrm{Mortality in control}\;\left(\%\right)/100\\ -\mathrm{Mortality in control}\;\left(\%\right)\times 100 \end{array}$$

### 2.4 Strain characterization

Strains with activity were observed on LB plates. Their microstructures were further observed using scanning electron microscopy. Their DNA was extracted using SDS-CTAB (Mesapogu et al., 2012), amplified by PCR using primers 27F (5′-AGAGTTTGATCCTGGCTC-3′) and 1492R (5′-CGGCTACCTTGTTACGACTT-3′) for the 16S rRNA gene, and sequenced by Sheng gong Bioengineering Co. (Shanghai, China). The EzBioCloud database^1^ was utilized for comparison. The 16S rRNA gene sequences of published strains with high similarity were used to construct phylogenetic trees using MEGA 5.05 software to determine the taxonomic status of the strains (Yoon et al., 2017).

### 2.5 Whole-genome sequencing (WGS) and antiSMASH

Strains were inoculated into LB broth and incubated at 30°C with shaking at 120 rpm for 24 h. Bacteria were pelleted by centrifugation at 10,000 rpm for 5 min at 4°C and then sent to Personal Bio (Shanghai, China) for sequencing. Personal Bio performed whole-genome shotgun sequencing (shotgun, paired-end WGS) using the Illumina NovaSeq platform. Data with removed adapter sequences were assembled *de novo* using A5-MiSeq and SPAdes software to construct contigs and scaffolds. Corrections were made using Pilon software (Bankevich et al., 2012; Coil et al., 2014; Walker et al., 2014). Functional annotations were obtained by comparing the assembled sequences with the NR, eggnog, KEGG, and Swiss-Prot databases using BLAST software. Secondary metabolite synthesis genes were predicted using antiSMASH.^2^

### 2.6 Isolation and characterization of insecticidal compounds

Strains exhibiting activity were fermented in large batches. Guided by antiSMASH analysis, small-molecule compounds were isolated and subjected to a purification process comprising macroporous resin adsorption, ODS column chromatography, gel column chromatography, and high-performance liquid chromatography (HPLC), then analyzed by Nuclear Magnetic Resonance (NMR) spectroscopy of hydrogen nuclei (^1^H NMR), Nuclear Magnetic Resonance (NMR) spectroscopy of carbon nuclei (^13^C NMR), Heteronuclear Single Quantum Coherence spectroscopy (HSQC), and Heteronuclear Multiple Bond Correlation spectroscopy (HMBC) spectroscopy (CHAI et al., 2007; Wu et al., 2013). Larger candidates, such as proteins, were precipitated using ammonium sulfate, purified using gel filtration, and sequenced by liquid chromatography–tandem mass spectrometry (LC–MS/MS) (Li and Zhu, 2020; Koteshwara et al., 2021). Lipopeptides were ethanol precipitated and acid (HCl) precipitation was used to obtain lipopeptides (Alajlani et al., 2016), which were purified by three to five acid washes and deionized water rinses (at 5°C) in small amounts and many times to pH 6–7. Powdered lipopeptides were obtained by freeze-drying, analyzed using ACQUITY UPLC/VION IMS QTOF MS, and compositional testing was performed.

### 2.7 Insecticidal assays

Based on the results of the preliminary experiments testing the activity of the products against *A. gossypii*, gradient concentrations were prepared to conduct insecticidal activity tests. Toxicity regression curves were used to calculate lethal median con-centration (LC_50_) values (Paramasivam et al., 2017).

### 2.8 Biological safety assessment

*Danio rerio*, provided by Ding yuan Biotechnology (Alar) were selected with an average length between 3.0 and 4.0 cm. The test fish were acclimated indoors for 7 days, during which the natural mortality rate should be below 1% to ensure their adaptation to the experimental environment. Feeding was stopped 24-h before the start of the experiment, and no feeding was conducted throughout the entire experimental period. The test water was tapping water that had been stored and aerated to remove chlorine for more than 24-h, with a pH of (8 ± 0.1), dissolved oxygen content of (7.06 ± 0.4) mg/L, and water temperature of (21 ± 0.4)°C.

The experiment employed a semi-static method (Xiong et al., 2022), with insecticidal active sub-stance concentrations set at 0.1 mg/L, 1 mg/L, 10 mg/L, and 100 mg/L. Zebrafish were placed into test and control tanks for the experiment, and the chemical solution was re-placed every 24-h to maintain consistent environmental conditions. Each treatment group consisted of 10 zebrafish. Observations and records of zebrafish poisoning symptoms and mortality began 6 h after the start of the experiment. The fish were observed and the number of deaths recorded at 24, 48, 72, and 96-h, with dead fish being promptly removed during the observation period.

### 2.9 Statistical analysis

The experimental data were analyzed by analysis of variance (ANOVA) as well as calculation of LC_50_ using IBM SPSS Statistics 25 software. Graphs were prepared using Origin 2021 software.

## 3 Results

### 3.1 Screening identified two active *Bacillus* strains

**Figure 1:**
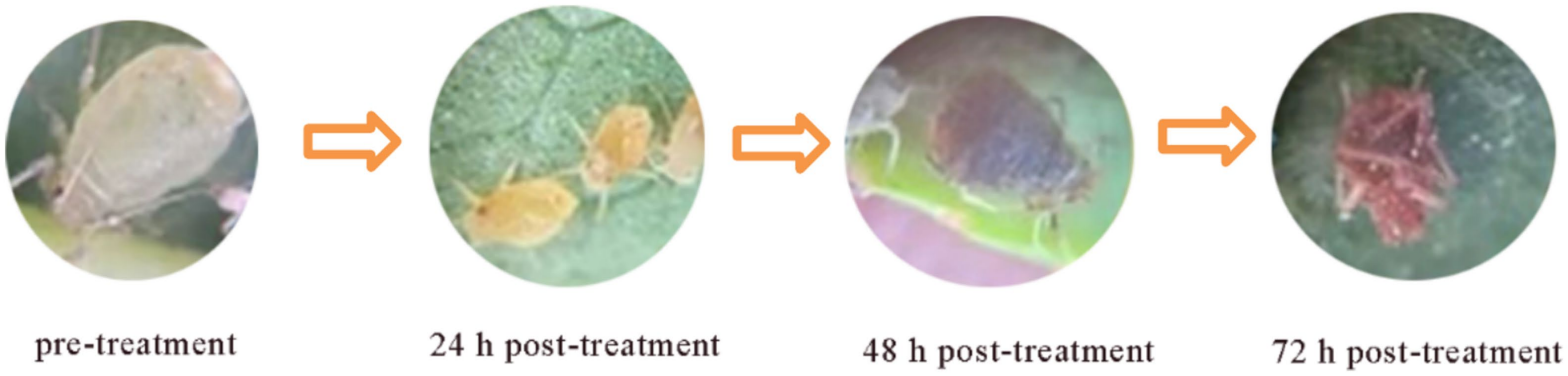
Photographs of antibacterial efficacy.

The leaf-dip method was used to determine the virucidal activity of shake flask fermentation broth of 107 strains of *Bacillus* spp. against *A. gossypii*. The corrected mortality rate of *A. gossypii* ≥ 60.00% after 48 h of treatment was used as the criterion for the evaluation of active strains. Two strains with high insecticidal activity were identified. Of the two, strain TRM82479 had higher activity, causing 75.00% mortality with a corrected mortality rate of 73.21% (Table 1). The efficacy of the bacterial strains’ fermentation broth against *Aphis gossypii* is shown in Figure 1. The screening results of the 107 candidate strains are shown in Supplementary Table S1.

**Table 1:** Strains with activity against *A. gossypii* obtained through screening.^1^

Strain	Most similar strain	Mortality rate (%)	Corrected mortality rate (%)
TRM82529	*Bacillus rugosus* SPB7	(50.00 ± 5.00) c	(46.43 ± 5.36) c
TRM82467	*Bacillus halotolerans* ATCC 25096	(71.67 ± 7.6) b	(69.64 ± 8.19) b
TRM82479	*Bacillus australimaris* NH71_1	(75.00 ± 5.00) b	(73.21 ± 5.36) b
Negative control	—	(6.67 ± 2.89) d	—
Chemical control	—	(76.67 + 2.89) a	(75.00 + 3.09) a

1 The table presents data as the mean ± standard deviation. Significant differences at the *p* < 0.05 level are denoted by distinct lowercase letters, as analyzed using Duncan’s new multiple range test.

As strain TRM82479 had higher insecticidal activity, it was subjected to species identification, WGS, antiSMASH, and isolation insecticidal active components.

### 3.2 Species identification

**Figure 2:**
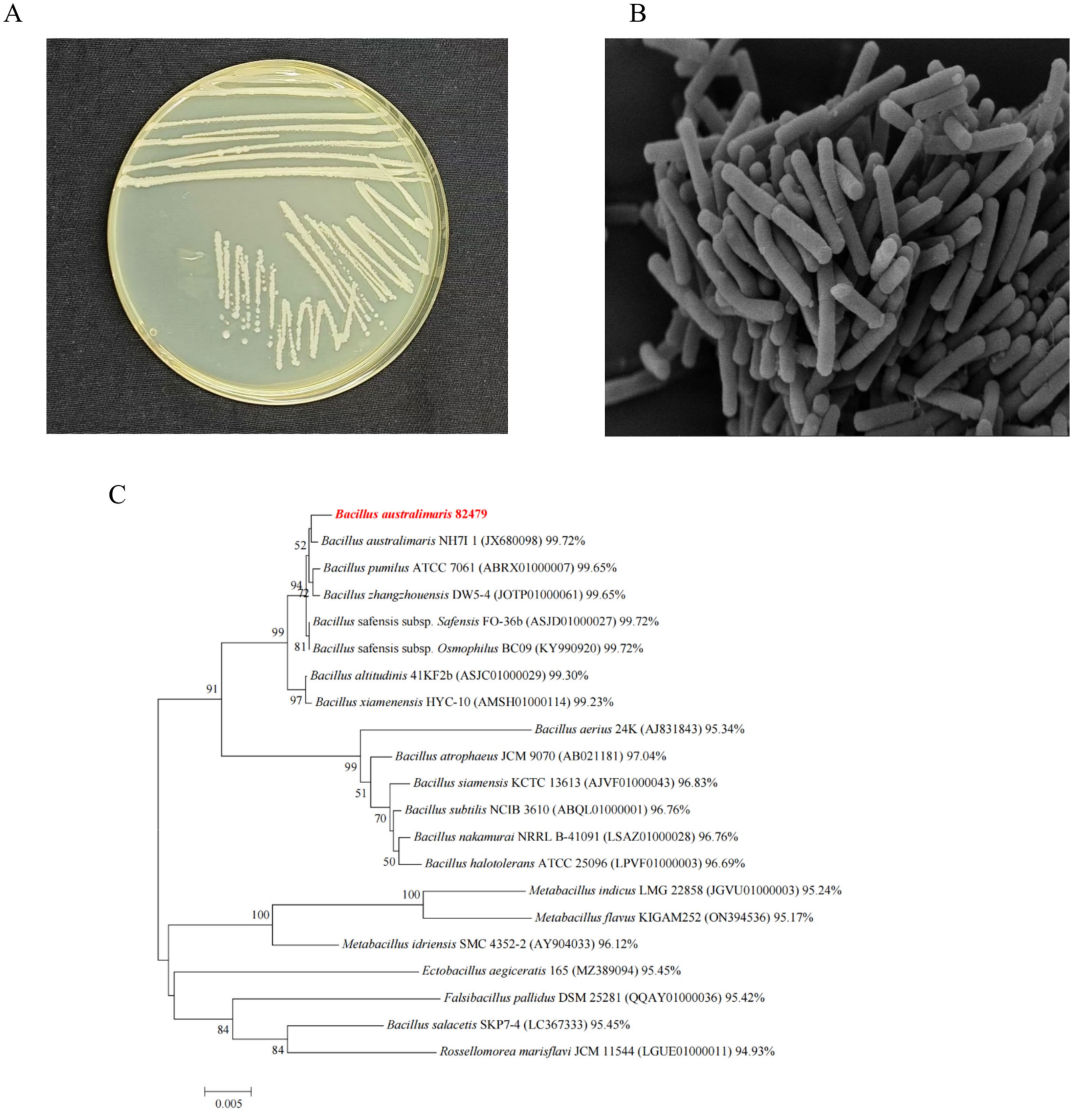
Species identification of strain TRM82479. **(A)** Colony diagram of strain TRM82479. **(B)** Scanning electron microscope image of strain TRM82479 (magnification 10,000). **(C)** Phylogenetic tree of strain TRM82479.

Strain TRM82479 formed milky-white, nearly round colonies on LB plates with a glossy surface (Figure 2A). By scanning electron microscopy, its cells were long and cylindrical, 2–4 μm in length, with a smooth cell surface (Figure 2B). The 16S rRNA sequence of strain TRM82479 was compared with those in the EzBioCloud database. We observed a similarity of 99.72% with the 16S rRNA sequence of *B. australimaris* NH71_1; other 16S rRNA sequences similar to this strain were retrieved and downloaded from the EzBioCloud database, and used to construct a phylogenetic tree. The tree suggests that TRM82479 is most closely related to *B. australimaris* NH71_1; therefore, the strain was preliminarily identified as *B. australimaris* TRM82479 (Figure 2C). The 16S rRNA sequence of this strain has been uploaded to NCBI under GenBank accession number PQ423094.

### 3.3 Strain TRM82479 genome contains clusters of genes associated with lipopeptide analog synthesis

**Figure 3:**
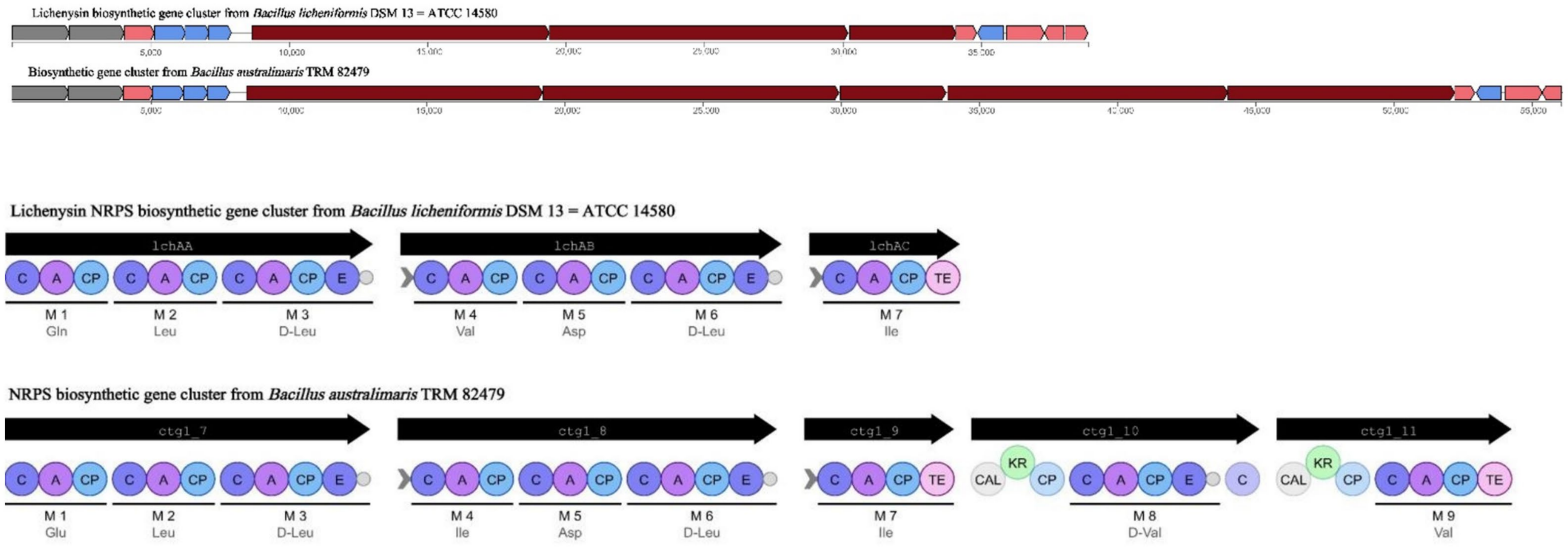
Cluster 8 vs. lichenysin synthesized gene clusters.

Based on WGS, 12 clusters of secondary metabolite synthesis genes were predicted using the antiSMASH database. Of these, six clusters had higher similarity to known gene clusters, with two associated with fengycin and lichenysin synthesis (Table 2). In a comparison of Cluster 8 with the lichenysin, 92.00% similarity was found. This cluster of includes three NRPS genes, lchAA, lchAB, and lchAC; it is responsible for the synthesis of a lipopeptide containing seven modules: Gln, Leu, D-Leu, Val, Asp, D-Leu, and Ile. In contrast, the cluster of *B. australimaris* TRM82479 includes multiple gene fragments (ctg1_7, ctg1_8, ctg1_9, ctg1_10, and ctg1_11) and contains nine modules: Glu, Ile, D-Leu, Ile, Asp, D-Leu, Ile, D, and Val. In modules 8 and 9, there were modifier enzyme domains such as CAL and KR, but the last putative esterase gene in the lichenysin biosynthesis gene cluster was not observed. These differences suggest that the TRM82479 lipopeptides are significantly different structurally and functionally from lichenysin (Figure 3). In summary, *B. australimaris* TRM 82479 may produce lipopeptides that are distinct from fengycin and lichenysin, and lipopeptides have been reported in recent years to have insecticidal activity, we isolated lipopeptides from this strain.

**Table 2:** The *B. australimaris* TRM82479 genome contains potential gene clusters involved in the production of secondary metabolites.

Clusters	Type	From	To	Most similar known clusters	Similarity
Cluster 1	Betalactone	6,442	34,091	Fengycin biosynthetic gene cluster from *B. velezensis* FZB42	53%
Cluster 2	Terpene	102,467	124,341	None	
Cluster 3	T3PKS	163,679	204,779	None	
Cluster 4	RiPP-like	513,619	523,945	None	
Cluster 5	Betalactone	663,643	696,094	None	
Cluster 6	NI-siderophore, terpene	184,353	212,695	Schizokinen biosynthetic gene cluster from Nostoc sp. PCC 7120 = FACHB-418r	60%
Cluster 7	RRE-containing	357,579	378,424	None	
Cluster 8	NRPS	165,368	249,092	Lichenysin biosynthetic gene cluster from *B. licheniformis* DSM 13 = ATCC 14580	92%
Cluster 9	RRE-containing, LAP	97,071	120,235	Plantazolicin biosynthetic gene cluster from *B. pumilus* ATCC 7061	91%
Cluster 10	Lanthipeptide-class-iii	25,195	47,885	None	
Cluster 11	Other	17,685	59,052	Bacilysin biosynthetic gene cluster from *B. velezensis* FZB42r	85%
Cluster 12	NRP-metallophore, NRPS	26,276	63,648	Bacillibactin biosynthetic gene cluster from *B. subtilis* subsp. subtilis str. 168	100%

### 3.4 Identification of insecticidal components

**Figure 4:**
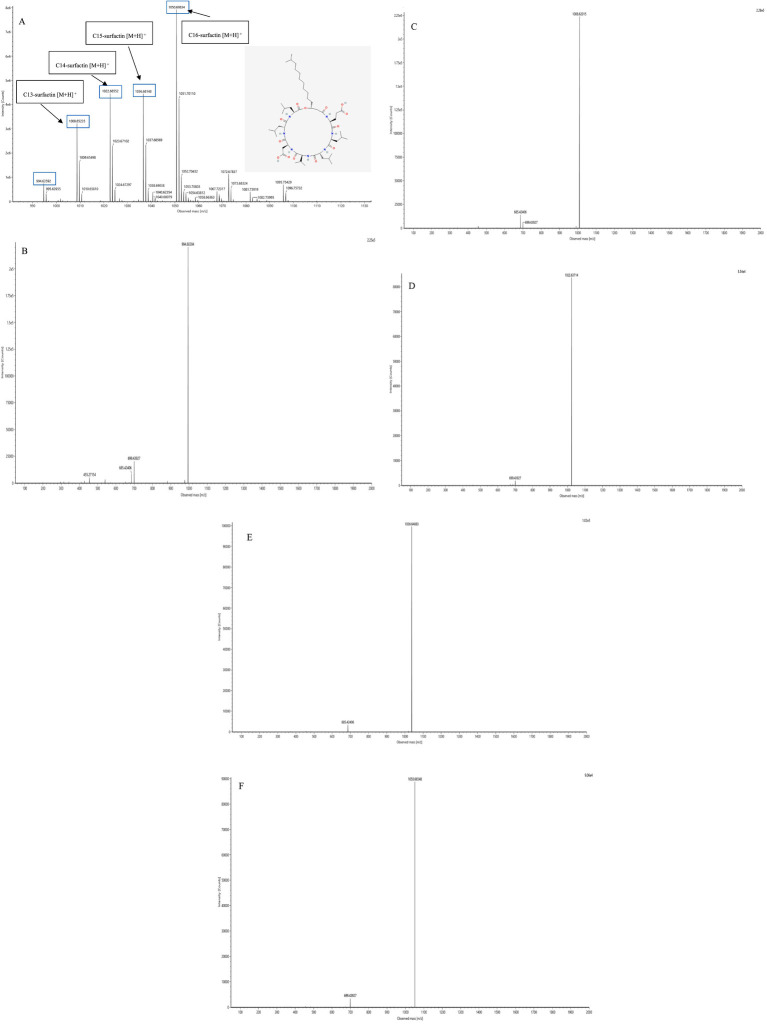
Plot of raw mass spectrometry data for surfactins. **(A)** Primary mass spectrum and two-dimensional structure of surfactins. **(B)** Mass: charge ratio (*m/z*) of 994.6 secondary mass spectra. **(C)** Mass: charge ratio (*m/z*) of 1,008.6 secondary mass spectra. **(D)** Mass: charge ratio (*m/z*) of 1,022.6 secondary mass spectrometry. **(E)** Mass: charge ratio (*m/z*) of 1,036.6 secondary mass spectra. **(F)** Mass: charge ratio (*m/z*) of 1,050.6 secondary mass spectra.

The lipopeptide compounds obtained by isolation and purification were detected by ACQUITY UPLC/VION IMS QTOF MS, and ion peaks with regularity of *m/z* of 994.63592, 1,008.65235, 1,022.66552, 1,036.68148, and 1,050.69834 appeared in the primary mass spectra (Figure 4A), which were presumed to be the fatty acid chains differing by one substituent methyl (–CH_2_) homologue of surfactins. The basic structure of surfactin is a cyclic lipopeptide formed by the combination of β-hydroxy fatty acids and a peptide containing seven amino acids through a lactone bond. The fatty acid chain length ranges from C_13_ to C_15_, and the most common sequence of the peptide is L-Glu → L-Leu → D-Leu → L-Val → L-Asp → D-Leu → L-Leu (Kowall et al., 1998; Price et al., 2007; Chen et al., 2008; Kim et al., 2010; Pecci et al., 2010; Ayed et al., 2014; Luo et al., 2014;Mnif et al., 2016). Secondary MS was performed on the above five ion peaks. Both 685.4 and 699.4 fragments were present in the secondary mass spectra. The fragment with a relative molecular mass of 685.4 is presumed to correspond to the fatty acid chain + Glu (the N-terminal fragment after ring-opening), while the fragment with a relative molecular mass of 699.4 is likely due to an increase in the fatty acid chain length by one CH_2_, or the replacement of Val with Leu or Ile. Together, these data strongly suggest that the lipopeptide analog belongs to the surfactin series (Figures 4B–F).

### 3.5 Surfactin insecticidal activity

**Figure 5:**
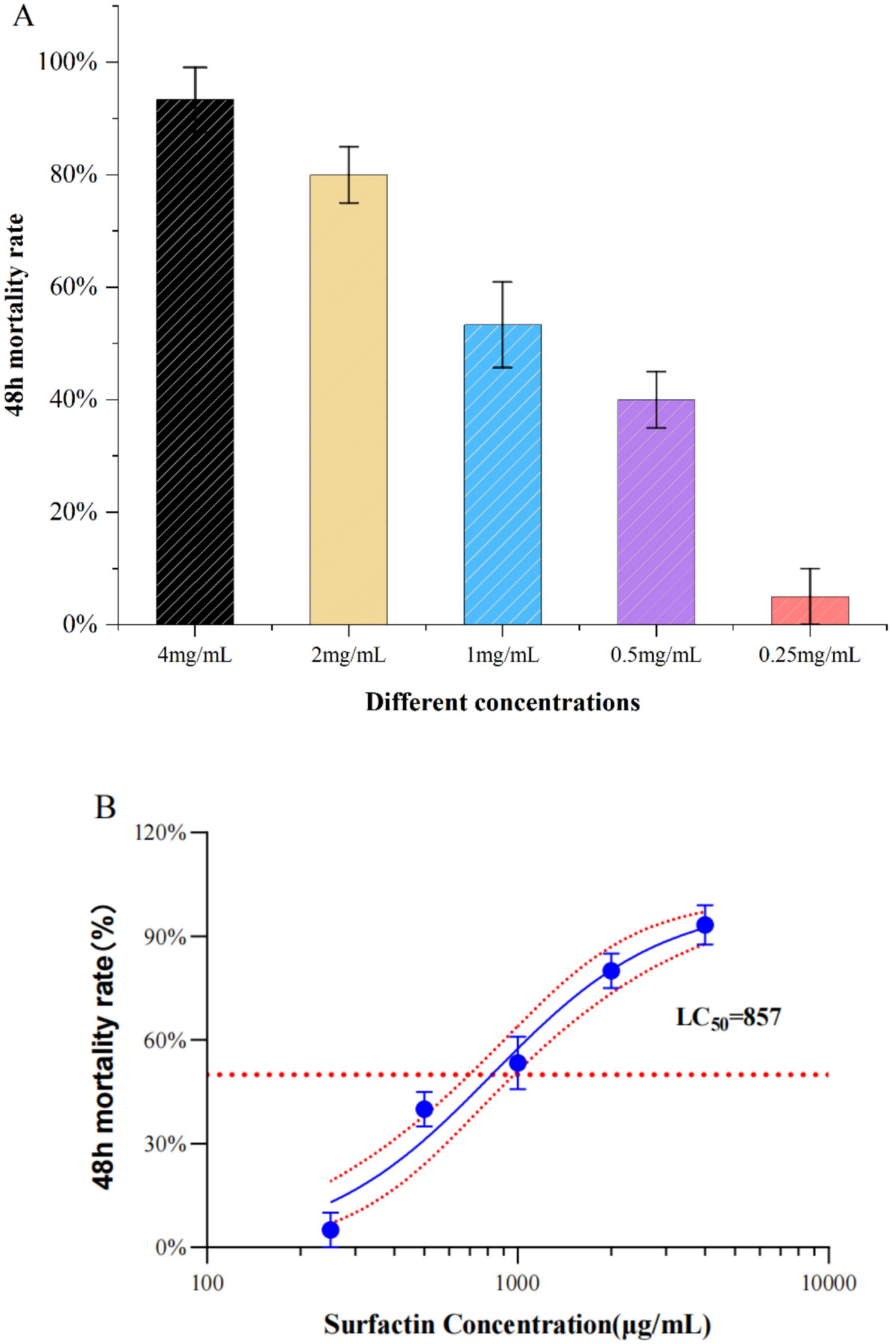
Graph of results of insecticidal activity of surfactins. **(A)** Effect of different concentration treatments on 48 h mortality of *A. gossypii* (48 h). **(B)** LC_50_ values of Surfactin’s 48 h virulence activity against *A. gossypii*. (The x-axis represents the surfactin concentration on a logarithmic scale ranging from 100 μg/mL to 10,000 μg/mL, where each division on the axis corresponds to a tenfold increase in concentration).

The aqueous surfactin was dissolved in deionized water with ultrasound assistance at a concentration of 4 mg/mL and tested for *A. gossypii* killing activity using foliar sprays (Paliwal et al., 2022), which yielded an *A. gossypii* mortality rate of 93.33% at 48 h. Surfactin at concentrations of 2 mg/mL, 1 mg/mL, 0.5 mg/mL, and 0.25 mg/mL yielded mortalities of 80.00, 53.33, 40.00, and 5.00%, respectively, (Figure 5A). The toxicity regression equation was calculated as *Y* = 2.331*X* + 0.156, with an LC_50_ of 0.857 mg/mL and an LC_95_ of 4.350 mg/mL (Figure 5B; Supplementary Table S2).

### 3.6 Surfactin biological safety

The toxicity of surfactin to zebrafish was evaluated using a semi-static method. At the 6-h point of the experiment, no mortality was observed in either the treatment or control groups. By the end of the experiment, the final mortality rate in the control group was 10%. The toxicity test results for surfactin indicated that, under a treatment concentration of 100 mg/L, the number of zebrafish deaths ranged from 0 to 2 between 24 and 96-h post-treatment, resulting in a final mortality rate of 20%. Consequently, it can be inferred that the LC_50_ value for surfactin produced by strain TRM82479 after 96-h of exposure to zebrafish is greater than 100 mg/L. According to the OECD standards for fish toxicity (Acute Category 1: 96-h LC_50_ ≤ 1 mg/L, Acute Category 2: 1 mg/L < 96-h LC_50_ ≤ 10 mg/L, Acute Category 3: 10 mg/L < 96-h LC_50_ ≤ 100 mg/L), the actual LC_50_ value measured for surfactin after 96-h of treatment was significantly higher than 10.0 mg/L. This suggests that surfactin produced by strain TRM82479 is of low toxicity to fish, posing a low safety risk.

## 4 Discussion

*A. gossypii* is one of multiple pests that pose a serious threat to cotton crops. Bacteria provide safer and more environment-friendly alternatives to commercially available synthetic insecticides (Ayilara et al., 2023). Numerous studies have shown that certain *Bacillus* species exert insecticidal effects against multiple pests and diseases in a wide range of crops (Islam et al., 2018; Pappas et al., 2021; Choi et al., 2023). In this study, we used 107 *Bacillus* strains isolated from the Taklamakan Desert to explore the *A. gossypii* killing potential. We identified two *A. gossypii*-killing strains, TRM82467 and TRM82479, under laboratory conditions, with strain TRM82479 showing a 48-h lethality of 75.00% against *A. gossypii* (Table 1). In addition, morphological observations revealed that *A. gossypii* was morphologically complete before the activity test, with a yellowish body color, smooth body surface, and clear antennae and foot structures; 24 h after the activity test, the color of *A. gossypii* became slightly darker, and the body surface appeared slightly wrinkled or discolored; 48 h after the activity test, the morphological changes of *A. gossypii* were more obvious, with significant wrinkling or discoloration of the body surface; severe crumpling (twisting of the abdomen and folding of the legs) and discoloration of *A. gossypii* worms occurred 72 h after the activity test (Figure 1). The hypothesis suggests that these alterations are induced by a synergistic interaction between microbial lipopeptides and extracellular cuticle-degrading enzymes, specifically chitinases and proteases. These enzymes target and degrade the chitin and protein constituents of the cuticle, thereby compromising the fundamental structural integrity and function of the exoskeleton (Akpor et al., 2021).

Previous studies have confirmed the insecticidal efficacy of multiple *Bacillus* species. Ruiu et al. found that the spores of *Brevibacillus laterosporu* infect *Musca domestica* and that this group of bacteria adsorbs secreted laterosporamine toxin to the spores, producing insecticidal activity (Ruiu et al., 2013). Fathy et al. isolated 200 strains of *B. subtilis* from mangrove ecosystems in Egypt and tested their activity against *Spodoptera frugiperda*. Among these, *B. subtilis* Esh73 had the highest larval mortality (80.00%) (Fathy et al., 2024). Ma et al. identified eight *B. thuringiensis* strains with activity against *Culex pipiens pallens* larvae and adults, with the spore-crystal mixture of strain A4 showing significant activity against larvae with an LC_50_ of 1.4 ± 0.5 μg/mL (Ma et al., 2023). Al-Azzazy et al. tested *B. subtilis* (2.470 × 10^8^ cfu/mL) and *B. qassimus* (3.320 × 10^8^ cfu/mL) under laboratory conditions on eggplant infested with *Tetranychus urticae* and reductions of 72.22 and 70.74%, respectively, after 7 days of treatment (Al-Azzazy et al., 2020). Liu et al. investigated the insecticidal activity of lipopeptides isolated from *B. velezensis* ZLP-101 against *Acyrthosiphon pisum*. The crude extract of ZLP-101 exhibited an LC_50_ of 411.535 mg/L against bean aphids. The active constituents identified in the extract encompassed iturins, engycins, surfactins, and spergualins (Liu et al., 2024).

This study analyzed the secondary metabolite synthesis genes of *B. australimaris* TRM82479 using antiSMASH and isolated lipopeptide analogs. The lipopeptides obtained from the isolation and purification were detected by ACQUITY UPLC/VION IMS QTOF MS and were confirmed to be a surfactin series by GNPS database comparison and literature research. Xia et al. reported that surfactin produced by *B. subtilis* YZ-1 was significantly toxic to *Tenebrio Molitor* by Xia et al. (2024). Moreover, there are few reports on the toxicity of surfactin against *A. gossypii*. Therefore, the findings of this study not only expand our understanding of the bioactive substances produced by the *B. australimaris* TRM82479 strain but also provide new scientific evidence for surfactin as a potential biopesticide.

## 5 Conclusion

Our findings indicate that *B. australimaris* TRM82479 and its produced surfactin series have significant potential for the green control of cotton aphids (*A. gossypii*). Currently, there are very few reports on the toxic activity of surfactin against cotton aphids. Surfactin, due to its high efficiency, low toxicity, and friendliness to non-target organisms, is expected to become an ideal alternative to traditional chemical pesticides. Our study has achieved significant results under laboratory conditions, but its effectiveness in field applications still requires further validation. In addition, future research can further optimize the fermentation production process of surfactin and explore its application effects in different crops and ecological environments to promote its widespread use in the agricultural field.

## Data Availability

The datasets presented in this study can be found in online repositories. The names of the repository/repositories and accession number(s) can be found at: https://www.ncbi.nlm.nih.gov/genbank/, PQ423094.

## Footnotes

^1^ www.EzBioCloud.net

^2^ antismash.secondarymetabolites.org
