# Supplementary material for: Surfactin: a novel Aphis gossypii killing surfactin produced by Bacillus australimaris TRM82479 of Taklamakan Desert origin
Source: Front Microbiol. 2025 Mar 12;16:1559495. doi: 10.3389/fmicb.2025.1559495 (PMC11936896; doi:10.3389/fmicb.2025.1559495)
Supplement: Supplementary file 1 [file Table_1.DOCX]

Supplementary Material

# Supplementary Tables

**Supplementary Table S1.** The information of the 107 strains of *Bacillus*.

| Strain No. | Maximum similar strains | Similarity | 48h mortality rate | 48h corrected mortality |
| --- | --- | --- | --- | --- |
| ck | *——* | —— | 6.67% | —— |
| Chemical control | *——* | —— | 76.67% | 75.00% |
| TRM58598 | *Bacillus wiedmannii* | 94.69 | 13.33% | 7.14% |
| TRM58416 | *Bacillus cereus* | 100 | 11.67% | 5.35% |
| TRM81953 | *Bacillus tequilensis* | 99.86 | 10.00% | 3.57% |
| TRM81956 | *Bacillus paralicheniformis* | 99.18 | 25.00% | 19.64% |
| TRM81961 | *Bacillus mesophilus* | 96.71 | 8.33% | 1.78% |
| TRM81969 | *Bacillus mediterraneensis* | 99.31 | 10.00% | 3.57% |
| TRM81979 | *Bacillus pumilus* | 99.39 | 18.33% | 12.50% |
| TRM81980 | *Bacillus mediterraneensis* | 95.47 | 13.33% | 7.14% |
| TRM81983 | *Bacillus mesophilus* | 97.19 | 10.00% | 3.57% |
| TRM81984 | *Bacillus mesophilus* | 98.95 | 11.67% | 5.35% |
| TRM81991 | *Bacillus mesophilus* | 98.08 | 6.67% | 0.00% |
| TRM81993 | *Bacillus tequilensis* | 99.93 | 10.00% | 3.57% |
| TRM81996 | *Bacillus mesophilus* | 98.95 | 6.67% | 0.00% |
| TRM82026 | *Bacillus zhangzhouensis* | 99.86 | 11.67% | 5.35% |
| TRM82057 | *Bacillus zhangzhouensis* | 99.45 | 20.00% | 14.28% |
| TRM82070 | *Bacillus zhangzhouensis* | 99.59 | 8.33% | 1.78% |
| TRM82075 | *Bacillus zhangzhouensis* | 99.32 | 16.67% | 10.71% |
| TRM82080 | *Bacillus velezensis* | 99.64 | 10.00% | 3.57% |
| TRM82082 | *Bacillus cheonanensis* | 99.37 | 15.00% | 8.93% |
| TRM82104 | *Bacillus zhangzhouensis* | 99.8 | 26.67% | 21.43% |
| TRM82110 | *Bacillus zhangzhouensis* | 98.98 | 10.00% | 3.57% |
| TRM82117 | *Bacillus salitolerans* | 96.85 | 8.33% | 1.78% |
| TRM82141 | *Bacillus mesophilus* | 96.46 | 16.67% | 10.71% |
| TRM82142 | *Bacillus velezensis* | 99.71 | 11.67% | 5.35% |
| TRM82143 | *Bacillus stercoris* | 99.93 | 31.67% | 26.78% |
| TRM82144 | *Bacillus mesophilus* | 96.33 | 8.33% | 1.78% |
| TRM82147 | *Bacillus swezeyi* | 98.91 | 10.00% | 3.57% |
| TRM82153 | *Bacillus infantis* | 99.59 | 15.00% | 8.93% |
| TRM82154 | *Bacillus velezensis* | 99.64 | 11.67% | 5.35% |
| TRM82155 | *Bacillus safensis subsp. safensis* | 99.51 | 11.67% | 5.35% |
| TRM82159 | *Bacillus atrophaeus* | 99.79 | 21.67% | 16.07% |
| TRM82172 | *Bacillus paralicheniformis* | 97.89 | 6.67% | 0.00% |
| TRM82179 | *Bacillus tequilensis* | 99.46 | 6.67% | 0.00% |
| TRM82183 | *Bacillus pumilus* | 99.14 | 8.33% | 1.78% |
| TRM82191 | *Bacillus albus* | 99.32 | 8.33% | 1.78% |
| TRM82193 | *Bacillus stercoris* | 99.93 | 10.00% | 3.57% |
| TRM82253 | *Bacillus mesophilus* | 98.5 | 11.67% | 5.35% |
| TRM82254 | *Bacillus tequilensis* | 99.73 | 8.33% | 1.78% |
| TRM82256 | *Bacillus cheonanensis* | 99.37 | 8.33% | 1.78% |
| TRM82264 | *Bacillus mesophilus* | 96.12 | 11.67% | 5.35% |
| TRM82273 | *Bacillus infantis* | 99.65 | 6.67% | 0.00% |
| TRM82279 | *Bacillus tequilensis* | 99.92 | 6.67% | 0.00% |
| TRM82282 | *Bacillus zhangzhouensis* | 99.93 | 8.33% | 1.78% |
| TRM82285 | *Bacillus stercoris* | 99.93 | 10.00% | 3.57% |
| TRM82308 | *Bacillus mesophilus* | 99.86 | 8.33% | 1.78% |
| TRM82321 | *Bacillus mesophilus* | 99.93 | 11.67% | 5.35% |
| TRM82330 | *Bacillus paralicheniformis* | 98.53 | 11.67% | 5.35% |
| TRM82334 | *Bacillus paralicheniformis* | 98.7 | 13.33% | 7.14% |
| TRM82337 | *Bacillus swezeyi* | 99.66 | 26.67% | 21.43% |
| TRM82341 | *Bacillus tequilensis* | 97.88 | 30.00% | 25.00% |
| TRM82350 | *Bacillus velezensis* | 98.87 | 6.67% | 0.00% |
| TRM82353 | *Bacillus dakarensis* | 99.43 | 8.33% | 1.78% |
| TRM82358 | *Bacillus salacetis* | 98.86 | 16.67% | 10.71% |
| TRM82361 | *Bacillus tequilensis* | 99.38 | 10.00% | 3.57% |
| TRM82362 | *Bacillus swezeyi* | 99.92 | 6.67% | 0.00% |
| TRM82363 | *Bacillus pumilus* | 99.11 | 10.00% | 3.57% |
| TRM82364 | *Bacillus mesophilus* | 98.41 | 11.67% | 5.35% |
| TRM82365 | *Bacillus mesophilus* | 98.07 | 40.00% | 35.71% |
| TRM82366 | *Bacillus swezeyi* | 99.59 | 33.33% | 28.57% |
| TRM82367 | *Bacillus mesophilus* | 96.35 | 41.67% | 37.50% |
| TRM82382 | *Bacillus mesophilus* | 97.65 | 6.67% | 0.00% |
| TRM82392 | *Bacillus dakarensis* | 99.25 | 6.67% | 0.00% |
| TRM82420 | *Bacillus tequilensis* | 99.8 | 13.33% | 7.14% |
| TRM82425 | *Bacillus stercoris* | 99.52 | 8.33% | 1.78% |
| TRM82442 | *Bacillus velezensis* | 98.29 | 8.33% | 1.78% |
| TRM82444 | *Bacillus stercoris* | 99.86 | 8.33% | 1.78% |
| TRM82459 | *Bacillus mesophilus* | 98.14 | 10.00% | 3.57% |
| TRM82466 | *Bacillus swezeyi* | 99.12 | 18.33% | 12.50% |
| TRM82467 | *Bacillus halotolerans* | 99.8 | 71.67% | 69.64% |
| TRM82473 | *Bacillus paralicheniformis* | 99.39 | 23.33% | 17.85% |
| TRM82475 | *Bacillus paralicheniformis* | 99.32 | 15.00% | 8.93% |
| TRM82479 | *Bacillus australimaris* | 99.66 | 75.00% | 73.21% |
| TRM82487 | *Bacillus paralicheniformis* | 99.66 | 8.33% | 1.78% |
| TRM82490 | *Bacillus pumilus* | 99.65 | 8.33% | 1.78% |
| TRM82493 | *Bacillus halotolerans* | 99.93 | 8.33% | 1.78% |
| TRM82494 | *Bacillus paralicheniformis* | 99.69 | 11.67% | 5.35% |
| TRM82495 | *Bacillus velezensis* | 97.14 | 23.33% | 17.85% |
| TRM82498 | *Bacillus paralicheniformis* | 99.93 | 11.67% | 5.35% |
| TRM82529 | *Bacillus rugosus* | 99.86 | 50.00% | 46.43% |
| TRM82535 | *Bacillus rugosus* | 99.63 | 16.67% | 10.71% |
| TRM82559 | *Bacillus swezeyi* | 99.37 | 20.00% | 14.28% |
| TRM82570 | *Bacillus paralicheniformis* | 99.61 | 16.67% | 10.71% |
| TRM82578 | *Bacillus tequilensis* | 99.79 | 15.00% | 8.93% |
| TRM82581 | *Bacillus australimaris* | 99.93 | 10.00% | 3.57% |
| TRM82595 | *[Bacillus cabrialesii](https://www.ezbiocloud.net/taxonomy?tn=Bacillus cabrialesii)* | 100 | 8.33% | 1.78% |
| TRM82599 | *Bacillus mesophilus* | 99.57 | 13.33% | 7.14% |
| TRM82604 | *Bacillus mesophilus* | 93.41 | 8.33% | 1.78% |
| TRM82606 | *Bacillus mesophilus* | 99.5 | 10.00% | 3.57% |
| TRM82608 | *[Bacillus mesophilus](https://www.ezbiocloud.net/taxonomy?tn=Bacillus mesophilus)* | 99.72 | 6.67% | 0.00% |
| TRM82617 | *[Bacillus mesophilus](https://www.ezbiocloud.net/taxonomy?tn=Bacillus mesophilus)* | 99.57 | 11.67% | 5.35% |
| TRM82619 | *Bacillus siamensis* | 99.72 | 20.00% | 14.28% |
| TRM82622 | *Bacillus suaedae* | 98.51 | 20.00% | 14.28% |
| TRM82623 | *Bacillus suaedae* | 97.95 | 11.67% | 5.35% |
| TRM82624 | *[Bacillus suaedae](https://www.ezbiocloud.net/taxonomy?tn=Bacillus suaedae)* | 99.09 | 25.00% | 19.64% |
| TRM82681 | *Bacillus velezensis* | 99.93 | 10.00% | 3.57% |
| TRM82682 | *Bacillus yapensis* | 95.95 | 8.33% | 1.78% |
| TRM82683 | *Bacillus haynesii* | 99.93 | 15.00% | 8.93% |
| TRM82684 | *Bacillus manusensis* | 99.79 | 8.33% | 1.78% |
| TRM82685 | *Bacillus paralicheniformis* | 99.79 | 10.00% | 3.57% |
| TRM82686 | *Bacillus licheniformis* | 99.5 | 6.67% | 0.00% |
| TRM82687 | *Bacillus tequilensis* | 99.93 | 6.67% | 0.00% |
| TRM82763 | *Bacillus licheniformis* | 95.53 | 8.33% | 1.78% |
| TRM82764 | *Bacillus licheniformis* | 99.57 | 23.33% | 17.85% |
| TRM82800 | *Bacillus licheniformis* | 99.78 | 8.33% | 1.78% |
| TRM82801 | *Bacillus licheniformis* | 98 | 10.00% | 3.57% |
| TRM82832 | *Bacillus licheniformis* | 99.58 | 35.00% | 30.35% |
| TRM82833 | *Bacillus licheniformis* | 99.86 | 20.00% | 14.28% |
