# Supplementary material for: Surfactin: a novel Aphis gossypii killing surfactin produced by Bacillus australimaris TRM82479 of Taklamakan Desert origin
Source: Front Microbiol. 2025 Mar 12;16:1559495. doi: 10.3389/fmicb.2025.1559495 (PMC11936896; doi:10.3389/fmicb.2025.1559495)
Supplement: Supplementary file 2 [file Table_2.DOCX]

Supplementary Material

# Supplementary Tables

**Supplementary Table S1.** Confidence level of surfactin in killing *A. gossypii*.

| **limit of confidence (math.)** | | | | | | | |
| --- | --- | --- | --- | --- | --- | --- | --- |
|  | probability | Concentration 95% Confidence Limit | | | 95% confidence limits for log(concentration)^b^ | | |
|  |  | estimation | lower limit | limit | estimation | lower limit | limit |
| PROBIT^a^ | .010 | .086 | .013 | .189 | -1.065 | -1.889 | -.724 |
|  | .020 | .113 | .021 | .229 | -.948 | -1.685 | -.640 |
|  | .030 | .134 | .028 | .260 | -.874 | -1.556 | -.585 |
|  | .040 | .152 | .035 | .286 | -.818 | -1.459 | -.544 |
|  | .050 | .169 | .042 | .308 | -.773 | -1.381 | -.511 |
|  | .060 | .185 | .049 | .330 | -.734 | -1.314 | -.482 |
|  | .070 | .199 | .055 | .349 | -.700 | -1.256 | -.457 |
|  | .080 | .214 | .063 | .368 | -.670 | -1.204 | -.434 |
|  | .090 | .228 | .070 | .387 | -.642 | -1.157 | -.413 |
|  | .100 | .242 | .077 | .404 | -.617 | -1.114 | -.393 |
|  | .150 | .308 | .116 | .488 | -.512 | -.936 | -.312 |
|  | .200 | .373 | .160 | .570 | -.428 | -.797 | -.244 |
|  | .250 | .440 | .209 | .654 | -.356 | -.680 | -.184 |
|  | .300 | .510 | .264 | .745 | -.292 | -.578 | -.128 |
|  | .350 | .586 | .327 | .846 | -.232 | -.486 | -.073 |
|  | .400 | .667 | .397 | .962 | -.176 | -.402 | -.017 |
|  | .450 | .757 | .474 | 1.099 | -.121 | -.324 | .041 |
|  | .500 | .857 | .559 | 1.266 | -.067 | -.253 | .102 |
|  | .550 | .970 | .652 | 1.473 | -.013 | -.185 | .168 |
|  | .600 | 1.101 | .755 | 1.738 | .042 | -.122 | .240 |
|  | .650 | 1.254 | .869 | 2.084 | .098 | -.061 | .319 |
|  | .700 | 1.438 | .996 | 2.550 | .158 | -.002 | .407 |
|  | .750 | 1.668 | 1.144 | 3.201 | .222 | .058 | .505 |
|  | .800 | 1.968 | 1.323 | 4.162 | .294 | .121 | .619 |
|  | .850 | 2.385 | 1.553 | 5.700 | .377 | .191 | .756 |
|  | .900 | 3.038 | 1.883 | 8.543 | .483 | .275 | .932 |
|  | .910 | 3.221 | 1.971 | 9.430 | .508 | .295 | .975 |
|  | .920 | 3.433 | 2.070 | 10.503 | .536 | .316 | 1.021 |
|  | .930 | 3.681 | 2.183 | 11.829 | .566 | .339 | 1.073 |
|  | .940 | 3.980 | 2.317 | 13.515 | .600 | .365 | 1.131 |
|  | .950 | 4.350 | 2.477 | 15.742 | .638 | .394 | 1.197 |
|  | .960 | 4.829 | 2.679 | 18.842 | .684 | .428 | 1.275 |
|  | .970 | 5.491 | 2.947 | 23.519 | .740 | .469 | 1.371 |
|  | .980 | 6.514 | 3.342 | 31.616 | .814 | .524 | 1.500 |
|  | .990 | 8.527 | 4.067 | 50.495 | .931 | .609 | 1.703 |
